# Clinical characteristics, healthcare use, and annual costs among patients with dystrophic epidermolysis bullosa

**DOI:** 10.1186/s13023-022-02509-0

**Published:** 2022-09-29

**Authors:** James A. Feinstein, Anna L. Bruckner, Benjamin Chastek, Amy Anderson, Juan Roman

**Affiliations:** 1 Department of Pediatrics, University of Colorado School of Medicine, 13123 E 16th Ave, Aurora, CO 80045 USA; 2 Department of Dermatology, University of Colorado School of Medicine, 13123 E 16th Ave, B570, Aurora, CO 80045 USA; 3 Optum, 11000 Optum Circle, Eden Prairie, MN 55344 USA; 4 Krystal Biotech, 2100 Wharton Street, Suite 701, Pittsburgh, PA 15203 USA

**Keywords:** Administrative claims, Bandages, Comorbidity, Cost of illness, Disease burden, Epidermolysis bullosa, Financial burden, Genetic skin diseases, Health resources, Skin and connective tissue diseases

## Abstract

**Background:**

Dystrophic epidermolysis bullosa (DEB) is a serious, ultra-rare, genetic blistering disease that requires a multidisciplinary care team and lifelong, proactive disease management. To organize and optimize care, we comprehensively examined diagnoses, healthcare use, and annual costs in patients with DEB across all healthcare settings.

**Methods:**

A retrospective study was performed using electronic health record (EHR) data from Optum Clinical Database (January 1, 2016, through June 30, 2020). Patients with an epidermolysis bullosa (EB) diagnosis between July 1, 2016, and December 31, 2019, with ≥ 6 months of baseline and 12 months of follow-up activity were included. Patients were stratified by EB type: recessive DEB (RDEB), dominant DEB (DDEB), DEB (type unknown), and EB unspecified. Demographics, comorbid conditions, and healthcare resource utilization were identified from EHR data. Cost of bandages and total medical costs (US$) were identified from linked claims data.

**Results:**

A total of 412 patients were included, classified as having DDEB (*n* = 17), RDEB (*n* = 85), DEB (type unknown; *n* = 45), and EB unspecified (*n* = 265). Mean age was 38.4 years, and 41.7% had commercial insurance coverage. The most common comorbidities were mental health disorders, malnutrition, and constipation. Rates of cutaneous squamous cell carcinoma ranged from 0% (DDEB) to 4.4% (RDEB). Prescriptions included antibiotics (56.6%), pain medications (48.3%), and itch medications (50.7%). On average, patients had 19.7 ambulatory visits during the 12-month follow-up, 22.8% had an emergency department visit, and 23.8% had an inpatient stay. Direct medical costs among patients with claims data (*n* = 92) ranged from $22,179 for EB unspecified to $48,419 for DEB (type unknown).

**Conclusions:**

This study demonstrated the range of comorbidities, multiple healthcare visits and prescription medications, and treatment costs during 1 year of follow-up for patients with DEB. The results underscore that the clinical and economic burden of DEB is substantial and primarily driven by supportive and palliative strategies to manage sequelae of this disease, highlighting the unmet need for treatments that instead directly address the underlying pathology of this disease.

**Supplementary Information:**

The online version contains supplementary material available at 10.1186/s13023-022-02509-0.

## Background

Dystrophic epidermolysis bullosa (DEB) is a serious, life-limiting, ultra-rare genetic disorder caused by mutations in the gene *COL7A1* (3p21.1) [1–3], which encodes the type VII collagen (COL7) protein [2, 4]. DEB is characterized by a plane of skin cleavage in the uppermost dermis just beneath the lamina densa and typically involves blistering followed by scarring of the skin and other mucosal membranes in response to minimal friction or trauma [4]. There are 3 major types of epidermolysis bullosa (EB), including EB simplex, junctional EB, and dystrophic EB (Kindler syndrome is a fourth rare form of EB); DEB accounts for 30% of the total population with EB [2, 4]. DEB is further classified as autosomal dominant (DDEB) or recessive (RDEB), with phenotypic expression dependent on having 1 allele of the *COL7A1* gene affected with a mutation producing a dominant negative effect for DDEB and both alleles affected leading to deficient or absent COL7 for RDEB [4, 5]. DEB prevalence has been estimated at 3 to 10 cases per million across a number of countries, but this range may reflect inherent underdiagnosis of milder cases [2, 6–8]. Although RDEB is considered the more severe form, phenotypic overlap exists between RDEB and DDEB, and genetic testing is guideline recommended to establish an accurate DEB diagnosis, categorize the subtype, and enable better disease management [4].

Recent US data on the burden of illness in patients with DEB are limited. However, prior studies of patients with DEB indicate significant associated morbidity and mortality. Common complications of DEB include bacterial infection and septicemia, malnutrition, anemia, esophageal strictures, nail dystrophy and loss, ophthalmic disorders, dental caries, pseudosyndactyly, constipation, itch, and pain, necessitating a multidisciplinary approach to management [5, 9–11]. DEB adversely impacts patients’ quality of life by interfering with activities of daily living, sleep, and participation in sports and other recreational activities [9, 12]. Compared with the general population, patients with DEB are at an increased risk for more frequent and aggressive cutaneous squamous cell carcinoma that arises within areas of long-term skin wounds or repeated scarring [13–15]. This aggressive cutaneous squamous cell carcinoma can lead to limb amputation, metastasis, and shortened life expectancy, particularly among patients with RDEB [5, 13, 16].

Presently, there are no approved curative or disease-modifying treatments available for DEB [11]. Management of DEB focuses on supportive and wound care, prevention of blistering and infection, symptomatic relief of pain and itch, and prevention, monitoring, and treatment of complications [17–21]. Wound care remains the cornerstone of treatment and includes the use of semi-occlusive, protective bandages to the affected area to decrease pain and reduce and prevent blistering, scarring, and infection [18–20, 22]. Dressing changes and wound care can be time consuming and expensive, making it burdensome to patients and caregivers [9, 19, 21]. For many patients with RDEB, wound care requires more than 4 h per day [9].

Due to the comprehensive and lifelong care needed, the clinical and economic burden can be high for patients and healthcare systems [23, 24]. An analysis of patient-level clinical and claims data across all settings of care is required to fully understand the current scope of clinical care and associated costs. Such data would enable healthcare systems to organize and optimize care for the most common and most severe aspects of DEB and to assess for changes in utilization and costs as new therapeutics and interventions are developed and implemented. Thus, we conducted a real-world study to comprehensively measure the clinical characteristics, healthcare use, and annual associated costs among patients with DEB across all settings of care.

## Results

### Sample size and follow-up time

A total of 765 patients had ≥ 1 medical claim with a diagnosis of DEB (*International Statistical Classification of Diseases and Related Health Problems* revision 10 [ICD-10] diagnosis code Q81.2) or of less specific EB (Q81.8 or Q81.9) in the electronic health record (EHR) during the identification period (see Additional file 1). Of those, 412 had clinical activity in the EHR for the 6 months before the index date and 12 months after the index date, and 134 patients had ≥ 1 note containing a term of interest. Among the 412 patients, 92 were continuously enrolled in the claims database with medical benefits for the 12-month follow-up period. After reviewing the relevant physician notes and applying the anemia, gastrointestinal/stenosis, and syndactyly criteria, 17 patients were classified as having DDEB, 85 patients as having RDEB, 45 as having DEB (type unknown), and the remaining 265 as having EB unspecified. Among patients with available claims data, the cohort sample sizes were *n* = 5 (DDEB), *n* = 26 (RDEB), *n* = 5 (DEB [type unknown]), and *n* = 56 (EB unspecified).

### Demographics

Among all patients, the mean (standard deviation [SD]) age was 38.4 (25.7) years; age was lower among patients with RDEB and DDEB than among patients with DEB (type unknown) and EB unspecified (Table 1). With the exception of the RDEB cohort, most patients were 18 years of age or older. Across all cohorts, there were more females than males. Overall, 57.3% of patients were from the Midwest, 19.7% from the Northeast, 15.8% from the South, 4.1% from the West, and 3.2% from another region. Insurance type was commercial for 41.8% of patients, Medicare only for 15.8%, Medicaid only for 13.1%, a combination of insurance types for 16.5%, and unknown for 10.7%; 2.2% of patients were uninsured. Patients in the RDEB cohort were less likely to have commercial insurance and more than twice as likely to have Medicaid than patients in the other cohorts.

**Table 1:** Patient demographics (EHR data)

	DDEB (*n* = 17)	RDEB (*n* = 85)	DEB (type unknown) (*n* = 45)	EB unspecified (*n* = 265)
Age, years				
Mean (SD)	25.6 (23.5)	19.5 (14.8)	46.8 (25.9)	43.8 (25.4)
Median (IQR)	18 (13‒18)	16 (8‒28)	50 (21‒65)	47 (20‒65)
Age group, n (%)				
0‒10 years	4 (23.5)	30 (35.3)	4 (8.9)	33 (12.5)
11‒17 years	4 (23.5)	18 (21.2)	5 (11.1)	26 (9.8)
≥ 18 years	9 (52.9)	37 (43.5)	36 (80.0)	206 (77.7)
Female, n (%)	10 (58.8)	51 (60.0)	29 (64.4)	151 (57.0)
Region, n (%)				
Northeast	7 (41.2)	26 (30.6)	7 (15.6)	41 (15.5)
Midwest	9 (52.9)	36 (42.4)	25 (55.6)	166 (62.6)
South	1 (5.9)	17 (20.0)	9 (20.0)	38 (14.3)
West	0 (0.0)	1 (1.2)	4 (8.9)	12 (4.5)
Other	0 (0.0)	5 (5.9)	0 (0.0)	8 (3.0)
Insurance type, n (%)				
Commercial	8 (47.1)	24 (28.2)	17 (37.8)	123 (46.4)
Medicare	1 (5.9)	6 (7.1)	9 (20.0)	49 (18.5)
Medicaid	2 (11.8)	22 (25.9)	5 (11.1)	25 (9.4)
Uninsured	0 (0.0)	2 (2.4)	1 (2.2)	6 (2.3)
Other/unknown	6 (35.3)	17 (20.0)	3 (6.7)	18 (6.8)
Combinations	0 (0.0)	14 (16.5)	10 (22.2)	44 (16.6)

*DDEB* dominant dystrophic epidermolysis bullosa, *DEB* dystrophic epidermolysis bullosa, *EB* epidermolysis bullosa, *EHR* electronic health record, *IQR* interquartile range, *RDEB* recessive dystrophic epidermolysis bullosa, *SD* standard deviation

### Comorbidities

**Fig. 1:**
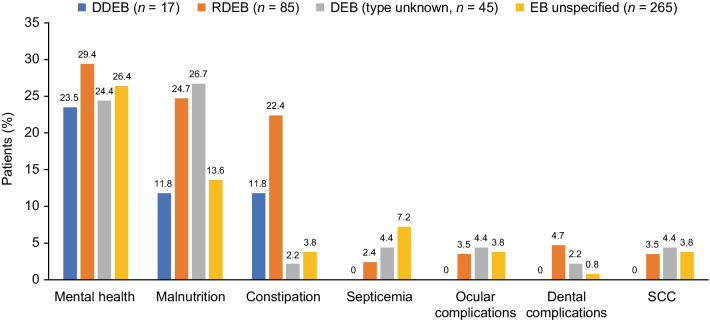
Percentage of patients with select comorbidities during the 12-month follow-up (EHR data). *DDEB* dominant dystrophic epidermolysis bullosa, *DEB* dystrophic epidermolysis bullosa, *EB* epidermolysis bullosa, *EHR* electronic health record, *RDEB* recessive dystrophic epidermolysis bullosa, *SCC* squamous cell carcinoma

The most common comorbidities or complications identified in the EHR during the 12-month follow-up were mental health disorders, malnutrition, and constipation (Fig. 1). Overall, 1.5% of patients were diagnosed with cutaneous squamous cell carcinoma in the EHR during the follow-up. The percentage of patients with cutaneous squamous cell carcinoma ranged from 0% in the DDEB cohort to 4.4% in the RDEB cohort. Among patients with pain scores (*n* = 218, 52.9%), on average, patients reported moderate pain (mean [SD] lowest to highest recorded values ranged from 1.3 [2.3] to 5.0 [3.7] out of 10).

### Medications

**Fig. 2:**
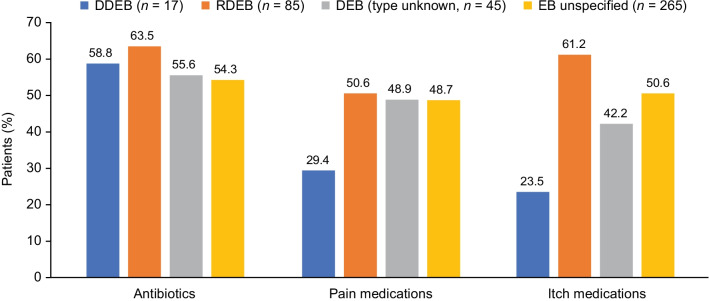
Percentage of patients with select medications during 12-month follow-up (EHR data). *DDEB* dominant dystrophic epidermolysis bullosa, *DEB* dystrophic epidermolysis bullosa, *EB* epidermolysis bullosa, *EHR* electronic health record, *RDEB* recessive dystrophic epidermolysis bullosa

During the 12-month follow-up period, 56.6% of the study population was prescribed or administered an antibiotic, 48.3% a pain medication, and 50.7% an anti-itch medication (Fig. 2). Similar overall rates of prescriptions filled were observed in the claims data among patients with available data (antibiotics, 57.6%; pain medications, 46.7%; anti-itch medications, 44.6%).

### Dermatologist visits

According to EHR data, 76.5% of patients with DDEB and 43.5% of patients with RDEB had ≥ 1 medical encounter with a dermatologist during the 12-month follow-up. In contrast, less than a third of patients with DEB (type unknown; 31.1%) and EB unspecified (25.7%) had an encounter with a dermatologist during the follow-up.

### Specialist visits of interest

**Fig. 3:**
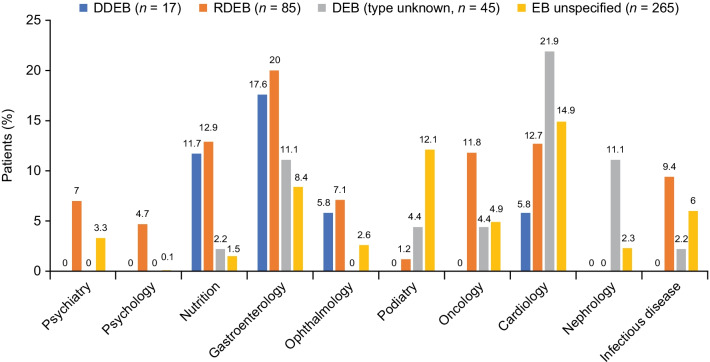
Percentage of patients with select specialty visits during the 12-month follow-up (EHR data). *DDEB* dominant dystrophic epidermolysis bullosa, *DEB* dystrophic epidermolysis bullosa, *EB* epidermolysis bullosa, *EHR* electronic health record, *RDEB* recessive dystrophic epidermolysis bullosa

Figure 3 shows the percentage of patients with a medical encounter with specialty providers of interest. Among these providers, the specialties visited included gastroenterology (11.9% overall), cardiology (11.9% overall), oncology (6.0% overall), and nutrition (4.4% overall). Notably, patients with RDEB were considerably more likely than those with DDEB to have mental health, oncology, and infectious disease visits during the 12-month follow-up period.

### Bandage use and costs

Bandage use in the EHR was documented for only 7 patients, who were all in the EB unspecified cohort. Thus, bandage use and cost were described based on medical claims data only. Bandage use was observed among a quarter (25.0% [23/92 patients]) of the sample with available claims data and was more common among patients with DDEB (60.0% [3/5 patients]), RDEB (42.3% [11/26 patients]), and DEB (type unknown; 40.0% [2/5 patients]) than among those with EB unspecified (12.5% [7/56 patients]). Among patients with bandage use, the median (interquartile range [IQR]) number of bandages per patient per 12 months was 24.0 (4.0‒55.0) overall and 31.0 (4.0‒52.0), 19.0 (6.0‒61.0), 93.5 (79.0‒108.0), and 24.0 (2.0‒54.0) in patients with DDEB, RDEB, DEB (type unknown), and EB unspecified, respectively. Mean (SD) annual total standardized cost (2019) of bandages (i.e., submitted as claims and approved) among patients with bandage use was $4705 ($13,202) overall and was higher for RDEB ($5341 [$14,082]) than for DDEB ($131 [$134]).

### All-cause healthcare utilization and costs

**Fig. 4:**
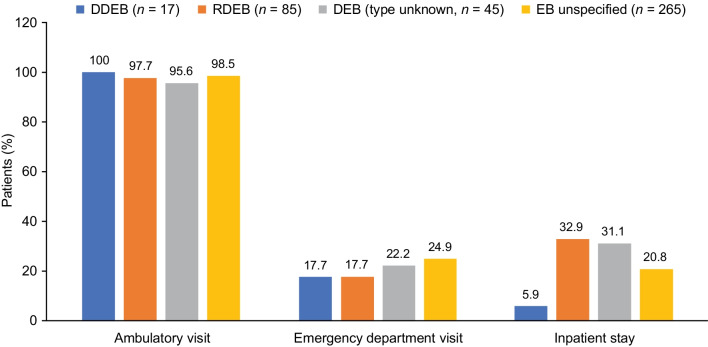
Percentage of patients with healthcare utilization during the 12-month follow-up (EHR data). *DDEB* dominant dystrophic epidermolysis bullosa, *DEB* dystrophic epidermolysis bullosa, *EB* epidermolysis bullosa, *EHR* electronic health record, *RDEB* recessive dystrophic epidermolysis bullosa

During the 12-month follow-up period, almost all patients (98.1%) had ≥ 1 ambulatory visit recorded in the EHR (Fig. 4). There was a median (IQR) of 12.0 (5.0‒26.0) ambulatory visits among all patients (Table 2). Overall, 22.8% of patients had an emergency department (ED) visit and 23.8% had an inpatient stay (Fig. 4). The mean (SD) number of ED visits per patient was 0.5 (1.8; Table 2). Markedly fewer patients with DDEB had an inpatient stay compared with other cohorts (Fig. 4). For those with an inpatient hospitalization, mean (SD) total days in the facility for all cohorts was 10.5 (23.1) per patient (Table 2). Among patients with claims data (*n* = 92), mean (SD) medical costs were $23,609 ($50,710) per patient (Table 3). According to claims data, 41.3% of patients overall (60.0% with DDEB, 65.4% with RDEB, 40.0% with DEB [type unknown], and 28.6% with EB unspecified) used home health services during the 12-month follow-up period. The mean (SD) number of days of home healthcare per patient was reported as 17.2 (54.7) overall, which was highest among patients with RDEB at 47.1 (96.2) days and lowest among patients with EB unspecified at 4.3 (11) days (associated costs reported in Table 3).

**Table 2:** Number of all-cause healthcare encounters during the 12-month follow-up (EHR data)

	DDEB (*n* = 17)	RDEB (*n* = 85)	DEB (type unknown) (*n* = 45)	EB unspecified (*n* = 265)
Ambulatory visits				
Mean (SD)	14.1 (14.0)	19.4 (23.3)	19.4 (23.3)	20.3 (23.9)
Median (IQR)	6.0 (5.0‒26.0)	12.0 (4.0‒28.0)	10.0 (5.0‒26.0)	13.0 (6.0‒26.0)
Emergency department visits				
Mean (SD)	0.2 (0.4)	0.7 (3.2)	0.4 (1.2)	0.5 (1.2)
Median (IQR)	0.0 (0.0‒0.0)	0.0 (0.0‒0.0)	0.0 (0.0‒0.0)	0.0 (0.0‒0.0)
Inpatient days (patients with inpatient stay)				
n	1	8	24	65
Mean (SD)	2.0 (–)	13.1 (39.9)	8.1 (5.9)	10.0 (12.2)
Median (IQR)	2.0 (2.0‒2.0)	4.0 (2.0‒8.0)	7.0 (3.0‒11.0)	5.0 (2.0‒10.0)

*DDEB* dominant dystrophic epidermolysis bullosa, *DEB* dystrophic epidermolysis bullosa, *EB* epidermolysis bullosa, *EHR* electronic health record, *IQR* interquartile range*, RDEB* recessive dystrophic epidermolysis bullosa, *SD* standard deviation

**Table 3:** All-cause healthcare costs (US$) during the 12-month follow-up^a^ (Market Clarity claims data)

	DDEB (*n* = 5)	RDEB (*n* = 26)	DEB (type unknown) (*n* = 5)	EB unspecified (*n* = 56)
Total costs				
Mean (SD)	31,836 (56,538)	29,995 (48,550)	50,482 (83,340)	26,501 (56,760)
Median (IQR)	4448 (1309‒21,610)	6882 (3272‒34,203)	20,635 (2515‒29,647)	9057 (933‒20,079)
Medical costs				
Mean (SD)	29,436 (55,422)	20,795 (35,142)	48,419 (84,352)	22,179 (53,736)
Median (IQR)	1667 (1175‒16,460)	5242 (2591‒23,407)	12,330 (2142‒28,534)	8082 (869‒15,410)
Home health costs^b^				
Mean (SD)	625 (1320)	7615 (20,374)	12,895 (28,685)	7219 (38,697)
Median (IQR)	37 (0‒104)	893 (0‒5132)	0 (0‒268)	0 (0‒13)
Pharmacy costs				
Mean (SD)	2400 (2289)	9200 (27,002)	2063 (3512)	4322 (12,976)
Median (IQR)	2781 (134‒3936)	89 (3‒2857)	524 (373‒1113)	262 (0‒2275)
Bandage costs				
Mean (SD)	131 (134)	5341 (14,082)	22,679 (31,792)	530 (939)
Median (IQR)	71 (37‒284)	472 (37‒2171)	22,679 (198‒45,159)	157 (45‒432)

Rounded to nearest dollar

*DDEB* dominant dystrophic epidermolysis bullosa, *DEB* dystrophic epidermolysis bullosa, *EB* epidermolysis bullosa, *IQR* interquartile range, *RDEB* recessive dystrophic epidermolysis bullosa, *SD* standard deviation

^a^Among patients with linked claims data

^b^Encompasses all services provided in the home setting (e.g., nursing services provided in the home, wound dressings, associated supplies)

## Discussion

The current analysis examined the clinical and economic burden of DEB in the United States. The most common complications were mental health disorders, malnutrition, and constipation. With the exception of mental health disorders (rates similar), complications were generally more frequent in patients with RDEB than in those with DDEB. Notably, cutaneous squamous cell carcinoma was observed at a rate 3.5 times higher in patients with RDEB than in the general population, which is consistent with previous studies demonstrating an increased risk of cutaneous squamous cell carcinoma in RDEB [7, 16]. Patients with DEB (type unknown), but not DDEB, had similarly elevated rates of cutaneous squamous cell carcinoma. Approximately three-quarters of patients with DDEB and half of those with RDEB had ≥ 1 medical encounter with a dermatologist over the past year. Patients with RDEB were generally more likely than those with DDEB to visit specialty care providers. Mean annual all-cause medical costs ranged from $20,795 in patients with RDEB to $48,419 in those with EB unspecified. These findings underscore the burden of cutaneous squamous cell carcinoma in this patient population, especially among patients with RDEB, and have several implications worth discussing in detail.

First, given the breadth of observed comorbidities and variety of care frequently utilized by patients with DEB, an interdisciplinary team approach (e.g., dermatologist, gastroenterologist, nutritionist, dentist, psychologist) is essential for efficiently coordinating, monitoring, and managing the care of patients with DEB [5]. The specialties frequently consulted in the 12-month follow-up period included gastroenterology (11.9% overall) and podiatry (8.5% overall). Compared with the DDEB cohort, greater percentages of patients with RDEB had mental health, gastroenterology, and oncology visits during the 12-month follow-up period; patients with RDEB were also observed to consult a nutritionist more often than those with DDEB. The higher rates of gastroenterology and oncology specialty visits among patients with RDEB are likely related to greater rates of malnutrition, constipation, and cutaneous squamous cell carcinoma in this patient cohort. Notably, the proportions of patients across cohorts with visits to psychiatry or psychology providers was low in relation to the reported rates of mental health comorbidities, suggesting that mental health conditions may be substantially undertreated in this patient population.

Second, by using a 360-degree-view comprising both EHR data and claims data, we were able to examine healthcare utilization and costs that further elucidate the total cost burden of DEB, including bandage and other diverse medical costs, to be up to $73,000 per patient per year. Patients with DEB had frequent ambulatory healthcare visits but relatively few ED visits per year, indicating that treatment was mostly provided on an outpatient basis. Among patients with hospitalizations, the mean length of stay was particularly high for patients with RDEB: 5 days longer than DEB (type unknown) and 11 days longer than DDEB. Unexpectedly, mean annual medical costs and total costs were lower for patients with RDEB than for those with DDEB, although the median costs were higher for patients with RDEB, suggesting that the mean costs in the DDEB group were likely skewed by one patient with high costs. However, the cost analyses were limited to patients with linked claims data and, thus, based on small sample sizes; the large SDs highlight the variability in cost data. Additionally, both the healthcare utilization and cost analyses were determined on an all-cause basis and may have been influenced by differences in age and insurance type between cohorts. Average annual pharmacy costs were 2- to 4-fold higher among patients with RDEB; however, again, interpretation of this finding is limited by the small sample sizes and high degree of variability.

Finally, bandaging-related costs impose a substantial financial impact on patients and their families. We estimated the mean annual cost of bandages paid for by insurance (not including out-of-pocket costs) among patients with bandage use to range from $131 for patients with DDEB to $22,679 for those with DEB (type unknown). The estimated annual cost was $5341 for patients with RDEB. However, the numbers of patients with bandage use claims in each cohort were very small. Because healthcare plans may not reimburse for the cost of bandages, such costs are challenging to capture in a patient population that is already small due to the rarity of the disease. Therefore, these results should be interpreted with caution.

These insurance-only bandage costs are less than previously reported total costs (insurance and out-of-pocket), which ranged annually from approximately $4000 to $47,000 for a neonate, $8000 to $99,000 for an infant, and $20,000 to $245,000 for a 10-year-old child in a study that estimated costs based on body surface area affected by wounds [19]. This difference suggests that the out-of-pocket costs of bandaging and wound care imposes a substantial out-of-pocket financial burden on patients with DEB and their families. Indeed, in a recently published survey of US patients with EB (or caregivers), 14% of patients with DDEB or DEB unspecified and 24% of those with RDEB reported monthly out-of-pocket dressing costs of $1000 or greater [24]. Approximately half of patients with DDEB or DEB unspecified and > 70% of those with RDEB indicated that their disease had a moderate or major financial impact. Further, approximately 65% of patients and caregivers reported that dressing and wound supplies were not reimbursed by a healthcare plan.

The results of this study must be viewed against potential limitations. First, the specific type of EB was defined by diagnosis codes and physician notes. Not all patients had notes, and those who did may not have had notes indicating the subtype. Therefore, some patients may have been misclassified as having EB unspecified because their provider did not contribute notes to the database. Second, a relatively small number of patients with DEB were included in the analyses, consistent with the rarity of this disease. Further, claims data were only available for 92 patients, which limits interpretation of the bandage and healthcare resource utilization measures. Although these results were limited to this patient sample and may not be broadly generalizable to other populations, our main data source, the EHR repository, is national in scope and contains enrollee data representative of the US national population. Third, claims data and EHRs may fail to capture complications and comorbidities that are not diagnosed. Also, the analysis of medication use is limited in that the presence of a claim for a filled prescription does not guarantee that the medication was consumed or taken as prescribed, and claims data do not capture medications filled over the counter or provided as samples by the physician or during a clinical trial. Fourth, these data may be biased toward patients who frequently seek care (i.e., reporting bias) and may have been less likely to capture healthcare utilization and costs for patients who rarely seek care or incur reimbursable expenses for their condition; thus, actual per-patient costs may be lower than represented by these data. Finally, the data sources used for this type of study do not always provide the granularity desired to fully capture the patient and family experience of DEB (e.g., patient-reported outcomes, informal caregiving, indirect costs), since several studies have shown that out-of-pocket costs and caregiver provision of informal care with associated loss of caregiver productivity/income are the major contributors to the economic burden of EB [9, 23, 24]. Therefore, additional prospective studies of important patient-reported outcomes obtained by directly surveying patients and caregivers are warranted. As healthcare systems, reimbursement schemes, and costs of materials vary considerably among different countries, studies employing uniform data collection methods to examine the healthcare utilization and costs associated with DEB across multiple countries would also be valuable.

## Conclusion

This study elucidates resource utilization and direct healthcare costs that patients incur across all settings of DEB care. The findings shed light on the devastating clinical impact of this disease, the substantial economic costs of the current supportive and palliative management strategies, and the dire need patients have for treatments that directly address the underlying pathology of this disease to reduce or even eliminate skin fragility and/or accelerate wound healing.

## Methods

### Data source and study design

This retrospective cohort study employed EHR, physician notes, and adjudicated medical claims data to select patients with a diagnosis of DEB in Optum’s Market Clarity database from January 1, 2016, through December 31, 2020. The Market Clarity database is a large repository of deidentified EHRs, physician notes, and administrative claims data for more than 101 million patients.

Baseline and follow-up measures were evaluated for patients with EB, stratified by the specific type of EB: RDEB, DDEB, DEB (type unknown), and EB unspecified (i.e., all unspecified EB diagnoses, including simplex and junctional), identified through ICD-10 codes. Physician notes with key terms relevant to the classification of RDEB, DDEB, and DEB (type unknown) were deidentified and reviewed to classify patients by subtype. The index date was defined as the date of first diagnosis of EB during the study identification period (July 1, 2016, through December 31, 2019), with priority given to the specific EB code over the nonspecific EB codes. The baseline period was 6 months prior to the index date, and the follow-up period was 12 months from or after the index date. Institutional Review Board (IRB) approval was obtained to review the deidentified physician notes. Patient privacy was preserved, and strict compliance with relevant Health Insurance Portability and Accountability Act data handling rules was maintained throughout.

### Study population

#### Inclusion criteria

To be included in the study, patients were required to have ≥ 1 medical claim with a diagnosis code for EB during the identification period. EB was identified from ICD-10 diagnosis codes specific to DEB (Q81.2) or for less specific EB (Q81.8 [other EB] or Q81.9 [EB, unspecified]). For patients with a diagnosis specific to DEB, the date of the first claim with a diagnosis of Q81.2 was considered the index date. For patients with claims for the less specific codes only, the earliest diagnosis of EB in the identification period was considered the index date. Patients were required to have clinical activity in the health system for 6 months prior to and 12 months from or after the index date.

#### Notes subset

Physician notes data were available for the subset of patients who sought care from a provider who contributed notes to the database. Notes were considered for review if they contained a keyword related the disease type, including: “dominant,” “recessive,” “homozygous,” and “heterozygous.” A complete list of relevant search terms is summarized in Additional file 2. Notes were deidentified and reviewed by Optum’s medical director to determine the patient’s disease type (RDEB, DDEB, DEB [type unknown], or EB unspecified). Following clinical review, patients were classified into cohorts based on the index diagnosis and notes classification. Patients were considered to have RDEB if they had a note indicating “recessive DEB” and were considered to have DDEB if they had a note indicating “dominant DEB,” regardless of their index diagnosis code. Those patients with a note indicating dominant and recessive DEB were included in the RDEB cohort only. Subsequently, the RDEB cohort was updated to also include patients who were: < 40 years of age and had anemia (anemia diagnosis, iron, erythropoietin, other erythropoietin agents, or blood transfusions and no diagnosis of cancer, beta thalassemia, or myelodysplastic syndrome); < 18 years of age and had stenosis, gastrostomy tube placement or nutrition supplements, and no diagnosis of hyperinsulinemia and hypoglycemia; or < 40 years of age and had a diagnosis of pseudosyndactyly or corrective hand or foot surgery. Patients with no notes indicating dominant or recessive DEB (including patients who did not meet additional criteria for RDEB described above) were classified as DEB (type unknown) if they had an index diagnosis of Q81.2 or had a note specifying DEB. All remaining patients were classified as EB unspecified. The EB unspecified cohort included patients with only EB diagnoses of Q81.8 or Q81.9 who either did not have notes available or had notes that did not indicate RDEB, DDEB, or DEB.

#### Claims data

Linked claims were available for a subset of the sample who were enrolled with a medical benefit. Medical claims or encounter data are collected from all available healthcare sites (inpatient hospital, outpatient hospital, ED, physician's office, surgery center, etc.) for virtually all types of provided services. A medical encounter included office visits, laboratory requests, ED visits, prescriptions, urgent care, inpatient visits, etc. Medical claims and coding conform to insurance industry standards. Medical claims included: multiple diagnosis codes recorded with ICD revision 9 (ICD-9) and ICD-10-Clinical Modification (ICD-10-CM) diagnosis codes; procedures recorded with ICD-9 and ICD-10-CM procedure codes, Current Procedural Terminology, or Healthcare Common Procedure Coding System codes; site of service codes; provider specialty codes; revenue codes (for facilities); standardized cost of service; and other information.

### Study variables

Variables evaluated using the EHR data included demographics, comorbid conditions, concomitant medications prescribed, dermatologist visits/encounters (including office visits, laboratory requests, ED visits, prescriptions, urgent care, inpatient visits, etc.), bandage use, and healthcare resource utilization. Patient demographics included age as of the index year, sex, region, and insurance type (commercial, Medicare [federally governed, with some components provided by private insurance companies], Medicaid [state governed], uninsured, unknown, or combinations of payers). Comorbid conditions were identified from ICD-9 and ICD-10 diagnosis codes and included septicemia, esophageal dilation, constipation, malnutrition, dental complications, ocular complications, surgeries (pseudosyndactyly), cutaneous squamous cell carcinoma, basal cell carcinoma, malignant melanoma, and mental health conditions. Concomitant medications were identified from National Drug Codes and Healthcare Common Procedure Coding System codes and included antibiotics, pain medications, and anti-itch medications. Healthcare resource utilization consisted of ambulatory visits (physician office and hospital outpatient visits), ED visits, inpatient admissions, specialty provider visits, and other medical services (laboratory and other nonlaboratory). Concomitant medications filled, bandage use, cost of bandages, and total medical cost (medical and pharmacy; US$) were identified from linked claims data among the subset of patients with available claims data. Costs were adjusted using the annual medical care component of the Consumer Price Index (CPI) to reflect inflation between the date of the claim and 2019, the most recent year for which the annual CPI was available.

### Statistical analysis

Patient characteristics and follow-up outcomes within each of the 4 cohorts (RDEB, DDEB, DEB [type unknown], and EB unspecified) were analyzed using descriptive statistics. Counts and percentages were calculated for categorical variables; medians, IQRs, means, and SDs were calculated for continuous variables. All analyses were conducted using SAS statistical software 9.4 (SAS Institute, Cary, NC, USA).

## Supplementary Information

**Additional file 1: Fig. S1.** Patient selection and categorization.

**Additional file 2: Table S1.** Note review keyword terms.

## Data Availability

The data found in our database contain proprietary elements owned by Optum and, therefore, cannot be broadly disclosed or made publicly available at this time. The disclosure of these data to third-party clients assumes certain data security and privacy protocols are in place and that the third-party client has executed our standard license agreement, which includes restrictive covenants governing the use of the data.

## Abbreviations

COL7: Type VII collagen
CPI: Consumer Price Index
DDEB: Dominant dystrophic epidermolysis bullosa
DEB: Dystrophic epidermolysis bullosa
EB: Epidermolysis bullosa
ED: Emergency department
EHR: Electronic health record
ICD-10 and ICD-9: International Statistical Classification of Diseases and Related Health Problems, 10th revision and 9th revision
IRB: Institutional review board
IQR: Interquartile range
RDEB: Recessive dystrophic epidermolysis bullosa
SD: Standard deviation
