# Supplementary material for: Clinical characteristics, healthcare use, and annual costs among patients with dystrophic epidermolysis bullosa
Source: Orphanet J Rare Dis. 2022 Sep 29;17:367. doi: 10.1186/s13023-022-02509-0 (PMC9524120; doi:10.1186/s13023-022-02509-0)
Supplement: Supplementary file 1 — Additional file 1: Fig. S1. Patient selection and categorization. [file 13023_2022_2509_MOESM1_ESM.pdf]

Additional file 1

Fig. S1 Patient selection and categorization.

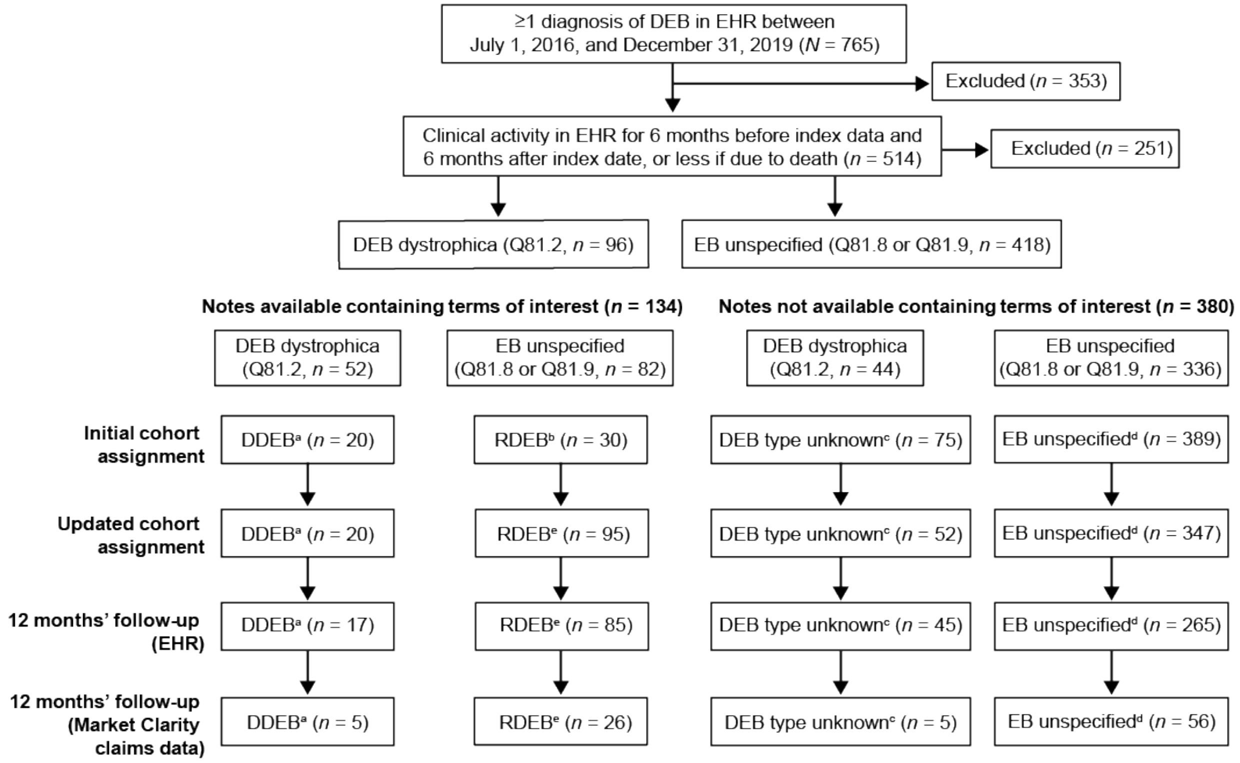

[a]All patients with a note indicating "Dominant" regardless of ICD classification.

[b]All patients with a note indicating "Recessive" or "Dominant and Recessive" regardless of ICD classification.

[c]Q81.2 and not in dominant or recessive assignment OR Q81.8/Q81.9 with note that indicates dystrophic and note does not indicate dominant or recessive category.

[d]Q81.8 or Q81.9, and patient has no notes or notes do not specify dominant, recessive, or dystrophic.

[e]All patients with a note indicating "Recessive" or "Dominant and Recessive" regardless of ICD classification. Patients in the dystrophic or unknown cohorts were reassigned if patient had anemia (anemia diagnosis, iron, erythropoietin, other erythropoietin agents, or blood transfusions and no diagnosis of cancer, beta thalassemia, or myelodysplastic syndrome, and was age < 40 years); had stenosis, gastrostomy tube placement or nutrition supplements and no diagnosis of hyperinsulinemia and hypoglycemia, and was age < 18 years; or had diagnosis of pseudosyndactyly and corrective hand or foot surgery, and was age < 40 years.

DDEB dominant dystrophic epidermolysis bullosa, *DEB* dystrophic epidermolysis bullosa, *EB* epidermolysis bullosa, *EHR* electronic health record, *ICD International Statistical Classification of Diseases and Related Health Problems*, *RDEB* recessive dystrophic epidermolysis bullosa
