# Supplementary material for: Clinical characteristics, healthcare use, and annual costs among patients with dystrophic epidermolysis bullosa
Source: Orphanet J Rare Dis. 2022 Sep 29;17:367. doi: 10.1186/s13023-022-02509-0 (PMC9524120; doi:10.1186/s13023-022-02509-0)
Supplement: Supplementary file 2 — Additional file 2: Table S1. Note review keyword terms. [file 13023_2022_2509_MOESM2_ESM.pdf]

Additional file 2

Table S1 Note review keyword terms

Recessive	Dominant	Autosomal
Genotype	Genome	Genotypic
Heterozygous	Homozygous	Trait
COL7A1	Alpha 1 type VII collagen	VII alpha 1
EBDCT	EBD1	EBR1
Long chain collagen	LC collagen	Dystrophic
